# Cases Showing the Constitutional Predisposition to the Formation of Schirrus in Different Parts of the Body at the Same Time

**Published:** 1827-02

**Authors:** 

**Affiliations:** Surgeon to St. George's Hospital.


### SCHIRRUS.

*Cases showing the Constitutional Predisposition to the Formation of Schirrus in different Parts of the Body at the same time.*

By Mr. Jeffreys, Surgeon to St. George's Hospital.

The constitutional predisposition to the formation of schirrous disorganisations in different parts of the body at the same time, is a pathological fact of great importance, as regards the question of operations for the removal of external tumors of this nature. The following cases, while they illustrate the general position, are also calculated to show how little, under such circumstances, is to be gained by the resources of art.

Case I.—Frances Bennett, forty-five years of age, was admitted into St. George's Hospital, April 21st, 1824, on account of a tumor in the right breast.

She stated, that seven years ago she first observed a lump on the outer side of the right breast. It was at that time about the size of a small nut, hard to the feel, and at times very painful; and it was loose and moveable under the skin. About Christmas, 1821, it had acquired the size of a hen's egg; and she then obtained admission into Guy's Hospital, where the tumor was extirpated by Sir A. Cooper. The wound healed very kindly, and she was discharged as cured in about six weeks. After the wound had cicatrised, and before she left the hospital, she perceived another small tumor, which had formed in the same breast near to the nipple. It caused her no uneasiness, and she therefore did not mention the circumstance to any body. At the end of a twelvemonth it began to give her pain, and to grow larger, and had continued gradually to increase in size and pain ever since.

At the time of her admission, the tumor was as large as an egg, and very painful and tender when touched. It was seated in the mammary gland, and felt hard, irregular, and schirrus-like, when examined. It was quite free and moveable over the pectoral muscle, but was attached to the superincumbent skin, which was discoloured; and the nipple was contracted and drawn in. Some of the absorbent glands in the axilla were slightly enlarged, but not at all indurated. Her general health appeared to be very good. The catamenia had ceased about two years.

The circumstances of the case were not of the most favourable nature; but she was desirous of having the part taken away, and, as nothing but the operation was calculated to afford her any chance of relief, the breast was amputated on the 26th of April. The glands in the axilla having subsided to nearly their natural size, were not meddled with. The diseased structure proved to be genuine schirrus. So much of the integuments had necessarily been taken away in the operation, that the cut edges could not be brought into apposition by at least half an inch. Nevertheless, the wound granulated and healed without any interruption, and she was discharged from the hospital on the 9th of June. At that time there was no sign of tumor existing in any other part of her body, and her general health was to all appearance excellent.

On the 22d of June of the following year (1825,) this poor woman was again admitted into the hospital, in the last stage of jaundice. She was much wasted and emaciated, and complained of occasional acute pain in the epigastric and hypogastric regions; and a perceptible enlargement and induration of the liver could be felt. Nothing checked the progress of the disease, and she sunk and died on the 19th of July.

*Dissection.*—The cicatrix left by the operation was quite healthy, and had acquired no adhesion to the subjacent parts. Several small, white, hard tubercles were found under the integuments covering the thorax, and a few in the pleura costalis. The lungs had no appearance of disease. There was a small quantity of serum effused into the cavity of the abdomen. The liver was enlarged, indurated, of a dark colour, and full of large white tubercles. Some of the mesenteric glands were enlarged and hardened; and a few tubercles were observed under the peritoneal covering of the intestines, and some on the bladder.

Case II.—Elizabeth Brown, a thin, spare woman, seventy years of age, was admitted May 24th, 1826. She had a schirrous tubercle, not much larger than a nutmeg, in the gland of the left breast. The skin over it was slightly discoloured, and adhering to the tumor; but there was no attachment to the parts beneath, the gland being free and moveable over the muscle. In the axilla was an indurated mass, as hard as a stone, filling up the space from the edge of the pectoralis major muscle to the clavicle. The arm was swollen and œdematous; and she complained of pain in the breast, axilla, shoulder, and arm.

She said, that she had not been at all aware of having any complaint in the breast until seven or eight months ago. About that time she met with a fall, and hurt her left shoulder. Soon afterwards, while rubbing the arm and shoulder, she discovered that she had a swelling in the axilla, and a lump in the breast.

Besides the disease in the breast, she complained of severe pain in the abdomen, which was rather more prominent than usual, and tender when pressed on, but neither tense nor hard. She complained also of great pain and uneasiness across the sacrum, and of irritation in making water. She was feeble and emaciated; had a furred tongue and costive bowels; and her pulse beat 100 in a minute. She was very low and dejected, and would hardly take any nourishment. The pain in the belly was increased by lying down, and she therefore generally sat up in bed, with her knees drawn up, and her chin on her breast.

The bowels were kept open by castor-oil; she was cupped on the sacrum, and had leeches to the abdomen; and she took opium and extract of hemlock to relieve the pain. Very little relief, however, was obtained; and she died on the 7th of June.

*Dissection.*—The tubercle in the breast, and the mass in the axilla, when cut into, presented the genuine character of true schirrus; the upper part of the latter was firmly attached to, and surrounded the axillary vessels. In the substance of the lungs of the right side were found two boney depositions, each about the size of a pea,

In that portion of the mesentery and mesocolon belonging to the termination of the ileum, the caput coli, and the beginning of the arch of the colon, was a large tumor, consisting of diseased glands, which exhibited the same kind of morbid structure as the tubercle in the breast. These parts were all matted and glued together into a confused mass, from the effects of former inflammation. One or two smaller specimens of the disease were found in other parts of the mesentery. Between the rectum and the sacrum was another tumor, as large as half an orange, having the same characters. It adhered firmly to the bone, and embraced the posterior half of the gut, so as considerably to diminish the size of its canal. In the edge of the left lobe of the liver was another tubercle, as large as an olive. There was no appearance of recent inflammation in the peritoneum or intestines.

Case III.—Amelia Hubbard, aged forty-four years, thin and delicate in her appearance, was admitted, October 11th, 1826, with a schirrous tumor in the right breast, as large as a duck's egg. It was situated in the mammary gland, and was hard and incompressible, and gave her a good deal of pain, particularly after being examined. The skin over it, as well as the nipple, which was shrunk and drawn in, adhered to the tumor. The integuments at that part were discoloured; and a little below the nipple were two superficial ulcerations, each about the size of a sixpence. The tumor had no attachment to the parts beneath it. Two or three absorbent glands in the axilla were enlarged and indurated; and there was another in the same condition above the clavicle. She complained of a good deal of pain darting through the breast, and had occasionally pain and numbness in the arm of the same side.

She said she had been sensible of having a lump in that breast for about two years, and that it had lately got larger and more painful. The catamenia had ceased four years.

On the seventh day after her admission, she was seized with violent rigors, that were followed by severe erysipelas in the diseased breast, side, and back; of which she died on the third day from the attack.

*Dissection.*—The tumor in the breast had the usual characters of scirrhous structure, and in the centre of the mass an imperfect suppuration had commenced. The glands in the axilla, and that above the clavicle, were converted into white hard tubercles. Within the thorax, immediately under the diseased breast, was a schirrous tumor, as large as half an orange, situated between the ribs and the pleura costalis, to both of which it was attached. The intercostal muscles, as well as the other parts intervening between this tumor and the breast, were free from any appearance of disease.

